# Disulfide engineering of the FANCM–MHF complex reveals constraints on crosslink design in symmetric oligomers

**DOI:** 10.1002/pro.70761

**Published:** 2026-08-17

**Authors:** Sho Ito, Tatsuya Nishino

**Affiliations:** ^1^ Faculty of Advanced Engineering Tokyo University of Science Tokyo Japan

**Keywords:** CENP‐SX, crosslinking specificity, disulfide engineering, DNA repair, FANCM, Fanconi anemia, MHF complex, protein crystallography, symmetric oligomer

## Abstract

The Fanconi anemia complementation group M protein (FANCM)–MHF complex is required for branched DNA recognition in the Fanconi anemia pathway, but structural analysis of the intact complex has been hindered by dissociation of FANCM from the FANCM‐associated histone fold (MHF) heterotetramer under crystallization conditions. Here, we used structure‐guided disulfide engineering to stabilize the FANCM–MHF interface and test whether local geometry is sufficient to predict crosslinking specificity in a symmetric oligomeric assembly. Using endogenous FANCM Cys759 as an anchor, we designed two MHF2 variants, Q74C and L77C. Both supported oxidation‐dependent crosslinking in the context of the FANCM–MHF complex, but with distinct outcomes. Q74C formed the intended FANCM–MHF2 disulfide, enabled crystallization of the intact heteropentamer, and preserved DNA‐binding behavior under the tested conditions. In contrast, L77C favored a competing MHF2‐MHF2 disulfide and yielded only the MHF heterotetramer after FANCM dissociation. Structural analysis further showed distinct crosslinking states for the two MHF tetramers in the asymmetric unit, consistent with local conformational heterogeneity at the MHF dimer–dimer interface. These results show that geometric plausibility alone does not predict crosslinking specificity in symmetric oligomers. Instead, symmetry‐related competing pathways can redirect the reaction toward an alternative assembly state. This study provides a practical route to stabilizing FANCM–MHF and reveals a key design constraint for engineered disulfides in symmetric multimeric assemblies.

## 1 INTRODUCTION

The Fanconi anemia (FA) pathway is a major genome maintenance system that coordinates the repair of DNA interstrand crosslinks and other replication‐associated lesions (Ceccaldi et al., 2016; Knipscheer et al., 2009). FANCM is a central component of this pathway and functions as an ATP‐dependent DNA translocase and scaffold that recognizes stalled replication forks and branched DNA structures and promotes recruitment of downstream repair factors (Meetei et al., 2005; Xue et al., 2015). A key determinant of FANCM function is its association with the histone‐fold proteins MHF1 and MHF2, also known as CENP‐S and CENP‐X, which together form the FANCM–MHF complex (Singh et al., 2010; Yan et al., 2010). FANCM‐associated histone fold (MHF) binding enhances FANCM DNA binding and contributes to recognition of diverse DNA substrates, including forked and Holliday junction (HJ)‐like structures (Coulthard et al., 2013; Fox et al., 2014; Xue et al., 2015; Zhao et al., 2014). Disruption of the FANCM–MHF interaction compromises the DNA damage response and genome stability (Singh et al., 2010; Yan et al., 2010).

Previous structural studies established that the C‐terminal region of FANCM associates with an (MHF1–MHF2)_2_ heterotetramer to form a heteropentameric complex, in which FANCM binds asymmetrically to MHF primarily through hydrophobic interactions (Ito & Nishino, 2021; Tao et al., 2012). Although this interface is sufficient to maintain the complex under native solution conditions, it is vulnerable to disruption by amphiphilic precipitants such as 2‐methyl‐2,4‐pentanediol (MPD) that are widely used in crystallization screens for protein–DNA complexes. Consequently, crystals grown from FANCM–MHF–DNA mixtures in our previous trials repeatedly contained only the MHF–DNA complex, indicating that FANCM dissociated during crystal growth (Ito & Nishino, 2021, 2022). The FANCM–MHF–DNA ternary complex, which would directly capture the mechanism of branched‐DNA recognition in the FA pathway, therefore remains structurally uncharacterized.

Covalent stabilization by an engineered disulfide bond offers a potential solution, provided the linkage preserves the overall architecture and function of the native assembly. In this study, we developed a structure‐guided disulfide engineering strategy centered on the endogenous FANCM Cys759 residue, thereby avoiding the introduction of additional cysteines into FANCM. Among MHF2 residues near Cys759, we identified two candidate crosslinks that appeared geometrically plausible at the FANCM–MHF2 interface. Comparison of these closely related designs revealed that geometric feasibility alone is insufficient to ensure selective crosslink formation in a symmetric oligomeric assembly. Symmetry‐related competing pathways impose an important additional constraint: one variant selectively stabilized the intended FANCM‐containing heteropentamer, whereas the other was diverted into an alternative MHF2–MHF2 linkage at the dimer–dimer interface (Craig & Dombkowski, 2013; Dani et al., 2003; Dombkowski, 2003).

## 2 RESULTS

### 2.1 Persistent precipitant‐induced dissociation of FANCM‐motivated covalent stabilization

**FIGURE 1:**
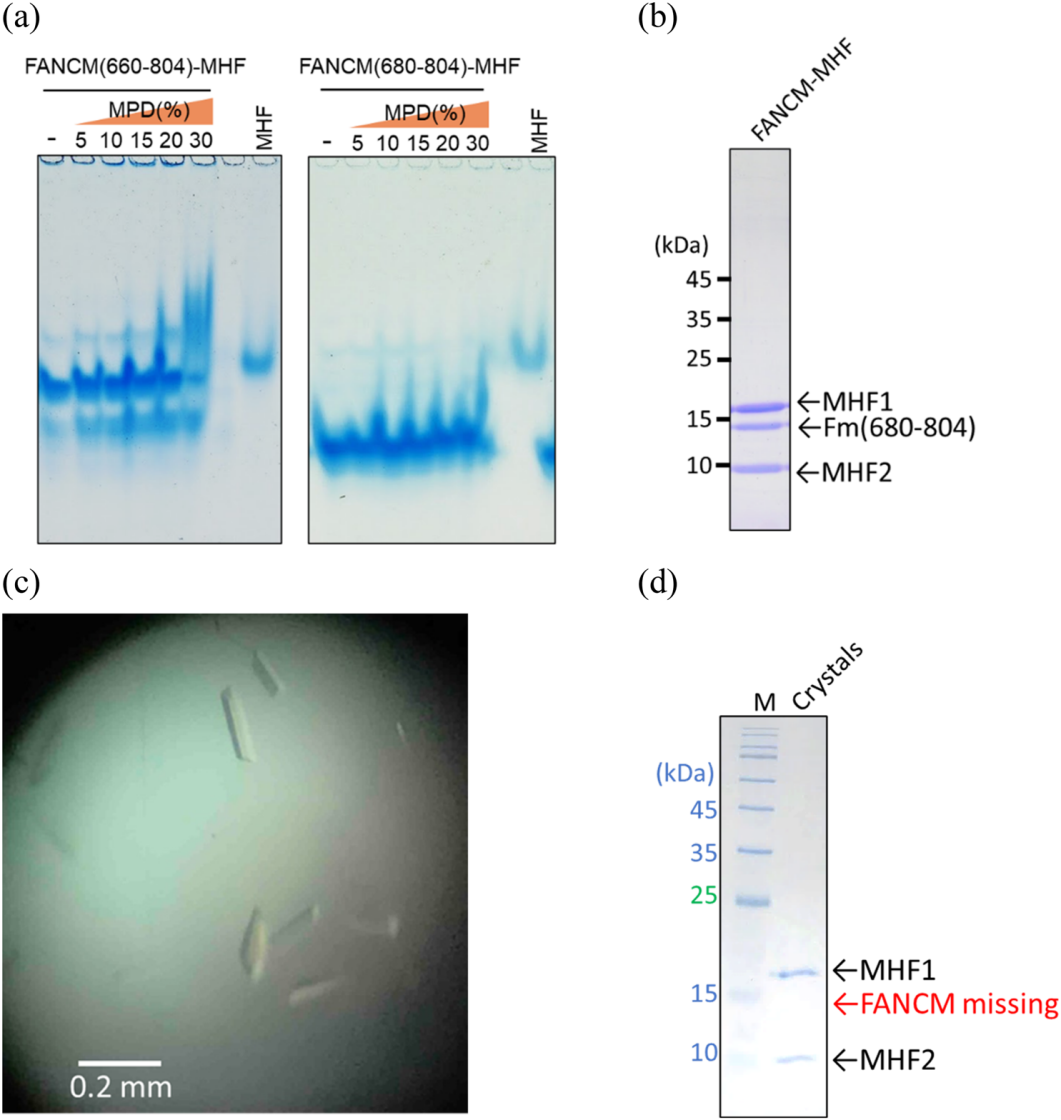
Construct optimization improves solution stability but does not prevent crystallization‐induced dissociation. (a) Native PAGE analysis of the original FANCM construct (residues 660–804) and optimized construct (residues 680–804) after incubation with 0%–30% (v/v) 2‐methyl‐2,4‐pentanediol (MPD) for 24 h at 4°C. FANCM–MHF and FANCM‐associated histone fold (MHF) were reconstituted using MHF1^ΔC^. The original construct shows multiple bands consistent with partial dissociation, whereas the optimized construct remains as a single major band. (b) SDS‐PAGE analysis of the final purified FANCM (680–804)‐MHF complex used for the FANCM–MHF‐DNA crystallization trial. FANCM–MHF were reconstituted using full‐length MHF1. (c) Representative crystal obtained from the optimized FANCM–MHF‐DNA crystallization trial. Scale bar: 0.2 mm. (d) SDS‐PAGE analysis of the dissolved crystal shown in (c). Only MHF1 and MHF2 are detected, whereas FANCM is absent, indicating that FANCM dissociated during crystallization. M: Protein molecular weight marker.

A major obstacle in our crystallization trials was dissociation of FANCM from the FANCM–MHF complex under MPD‐containing conditions. In our previous structural studies of the chicken FANCM–MHF complex, we found that the heteropentamer, consisting of one FANCM subunit (residues 660–804) bound to an MHF tetramer, was prone to dissociation in the presence of amphiphilic precipitants such as MPD (Ito & Nishino, 2021). To improve complex stability, we optimized the FANCM construct by removing the disordered N‐terminal region (residues 660–679). To compare the stability of the original and optimized constructs, both complexes were incubated with 0%–30% (v/v) MPD for 24 h at 4°C and analyzed by native polyacrylamide gel electrophoresis (PAGE). The original construct showed multiple bands consistent with partial dissociation, whereas the optimized construct remained as a single band, indicating improved stability in the presence of MPD (Figure 1a, left and right). The composition of each native PAGE band was confirmed by excision and sodium dodecyl sulfate–polyacrylamide gel electrophoresis (SDS‐PAGE) analysis, as described previously (Ito & Nishino, 2021); the lower band contained FANCM, MHF1, and MHF2, whereas the upper band contained MHF1 and MHF2 alone, confirming that the additional bands in the original construct corresponded to FANCM‐dissociated species (Figure 1a, right). SDS‐PAGE analysis of the final purified optimized complex showed the presence of FANCM, MHF1, and MHF2, consistent with formation of the intended heteropentameric complex (Figure 1b). Crystallization of the optimized FANCM–MHF complex with DNA yielded crystals similar to those obtained with the original construct (Figure 1c). However, analysis of dissolved crystals detected only MHF1 and MHF2, whereas FANCM was absent (Figure 1d), indicating that FANCM dissociated during crystallization. This result was consistent with our previous observations under MPD‐containing conditions (Ito & Nishino, 2021), suggesting that removal of the disordered region improved short‐term stability but was insufficient to preserve the FANCM–MHF interface during crystallization in the presence of organic solvent.

### 2.2 Structure‐guided disulfide design identified candidate crosslinks around endogenous FANCM Cys759

**FIGURE 2:**
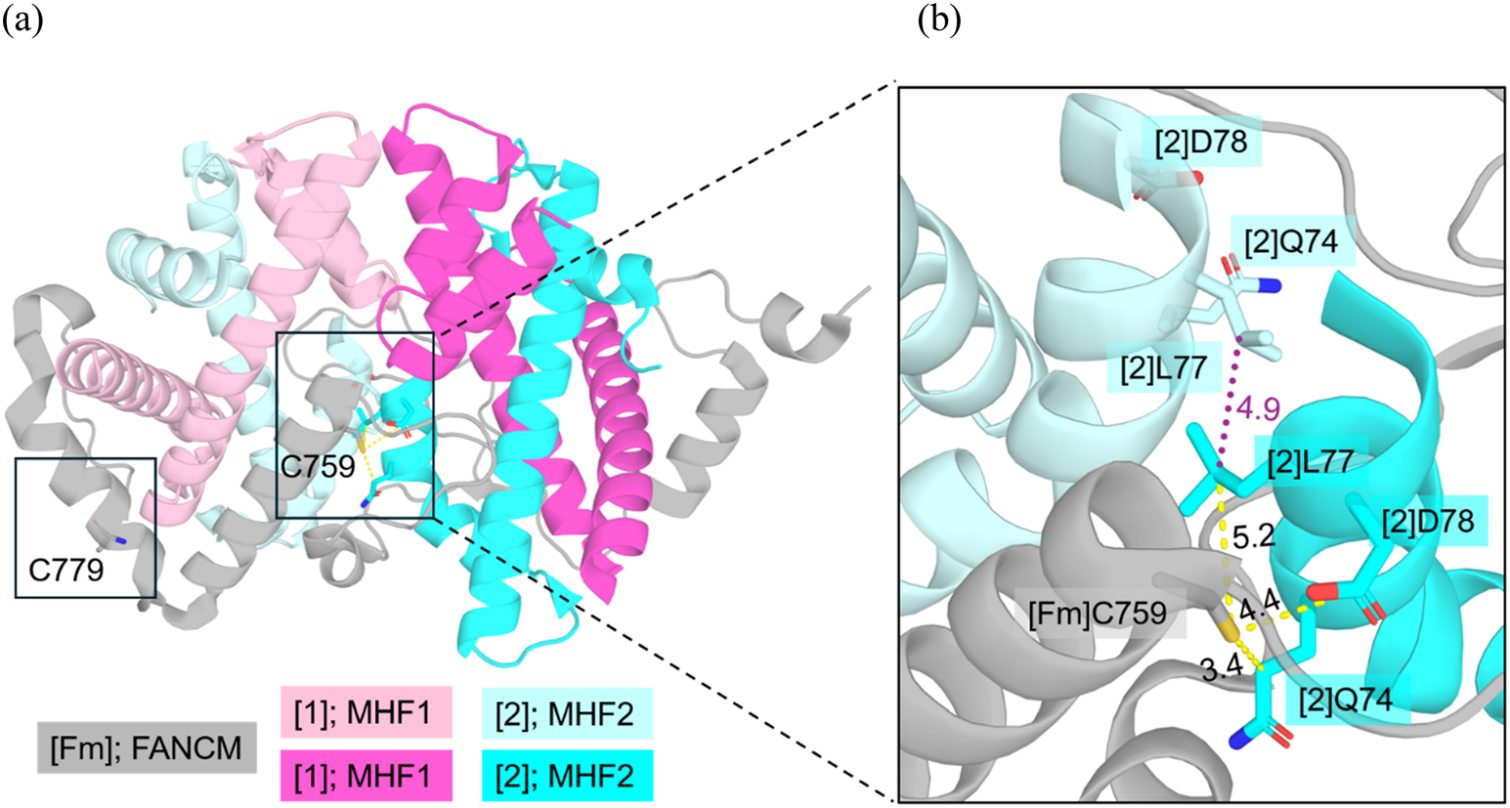
Endogenous FANCM Cys759 guided minimal disulfide design at the FANCM–MHF interface. (a) Overall structure of the FANCM–MHF heteropentamer (PDB entry 7DA2). FANCM is shown in gray, MHF1 in magenta (pale and dark), and MHF2 in cyan (pale and dark). The positions of the two endogenous cysteines retained in the optimized FANCM construct, Cys759 and Cys779, are indicated. Cys759 is located at the FANCM–MHF2 interface, whereas Cys779 is positioned on the opposite face of FANCM and away from the FANCM‐associated histone fold (MHF) binding surface. (b) Close‐up view of the FANCM–MHF2 interface around endogenous FANCM Cys759. MHF2 residues Gln74, Leu77, and Asp78 located within 4 Å of Cys759 are shown as sticks. Distances from the Sγ atom of FANCM Cys759 to the corresponding side‐chain carbon atoms of these residues are indicated. Gln74 and Leu77 were selected for experimental evaluation, whereas Asp78 was excluded because of unfavorable geometry.

**FIGURE 3:**
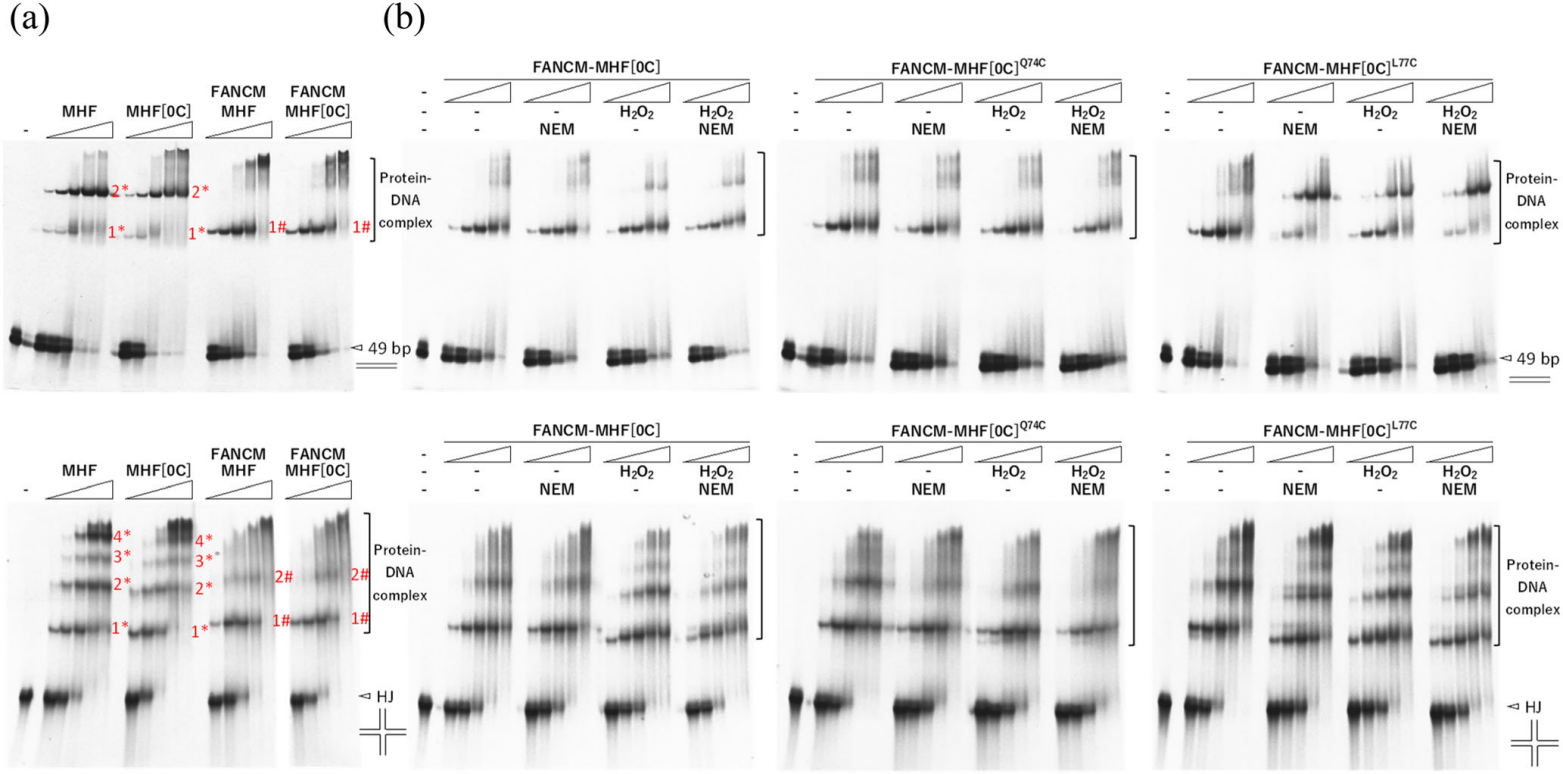
The Q74C‐stabilized complex retains DNA‐binding behavior under the tested conditions. (a) Electrophoretic mobility shift assays comparing FANCM‐associated histone fold (MHF), wild‐type FANCM–MHF, and FANCM–MHF reconstituted with MHF1[0C], using a 49 bp duplex (upper) and a Holliday junction substrate (lower). Major shifted bands are numbered in red. For the 49 bp duplex substrate: 1* and 2* for MHF; 1# for FANCM–MHF. For the Holliday junction substrate: 1*–4* for MHF; 1# and 2# for FANCM–MHF. (b) Electrophoretic mobility shift assays of FANCM–MHF[0C], FANCM–MHF[0C]^Q74C^, and FANCM–MHF[0C]^L77C^ complexes in the absence (−) or presence of *N*‐ethyl maleimide (NEM) or H_2_O_2_ followed by NEM treatment, using a 49 bp duplex (upper) and a Holliday junction substrate (lower). Major shifted bands are numbered in red (1*‐4* for MHF; 1#, 2# for FANCM–MHF).

To stabilize the FANCM–MHF interface by disulfide crosslinking, we sought to exploit endogenous cysteine residues within the FANCM construct rather than introduce new cysteines into both interacting partners. The optimized FANCM construct (residues 680–804) retains two endogenous cysteines: Cys759 and Cys779. Of these, Cys759 is located at the FANCM–MHF2 interface and does not form a covalent linkage in the native structure (Figure 2a). By contrast, Cys779 is positioned on the opposite face of FANCM; the nearest MHF residue is MHF1 Lys44, with an Sγ–Cγ distance of 5.9 Å, indicating that it is not favorably positioned for interface‐directed disulfide formation. We therefore selected Cys759 as the anchor point for disulfide design. Manual inspection of the FANCM–MHF structure (Protein Data Bank [PDB] entry 7DA2) identified three MHF2 residues within 4 Å of FANCM Cys759: Gln74, Leu77, and Asp78 (Figure 2b). The distances between the Sγ atom of Cys759 and the Cγ atoms of each residue were 5.3, 3.9, and 5.4 Å, respectively. Each of these residues was computationally substituted with cysteine using the Build Mutants protocol in Discovery Studio, and the resulting geometries were evaluated. MHF2^Q74C^ showed the most favorable geometry, whereas MHF2^D78C^ appeared unfavorable because of steric strain and was excluded. Notably, Leu77 lies at the MHF tetramer interface, where the non‐crystallographic symmetry‐related Leu77 residues are separated by 4.9 Å, whereas Gln74 faces away from the symmetric partner. Based on these geometric evaluations, we prioritized MHF2^Q74C^ and MHF2^L77C^ for experimental validation. The contrasting positions of these two residues provided a comparison between two geometrically plausible designs with different susceptibility to symmetry‐related competition. To avoid ambiguity in crosslinking interpretation arising from endogenous cysteine residues in MHF1, all subsequent experiments were performed using MHF1[0C], a variant in which the three endogenous cysteines (Cys26, Cys28, and Cys55) were substituted with alanine. EMSA confirmed that MHF1[0C] retains DNA‐binding activity comparable to wild‐type MHF1 (Figure 3a).

### 2.3 Closely related cysteine designs produced distinct crosslinking specificities

**FIGURE 4:**
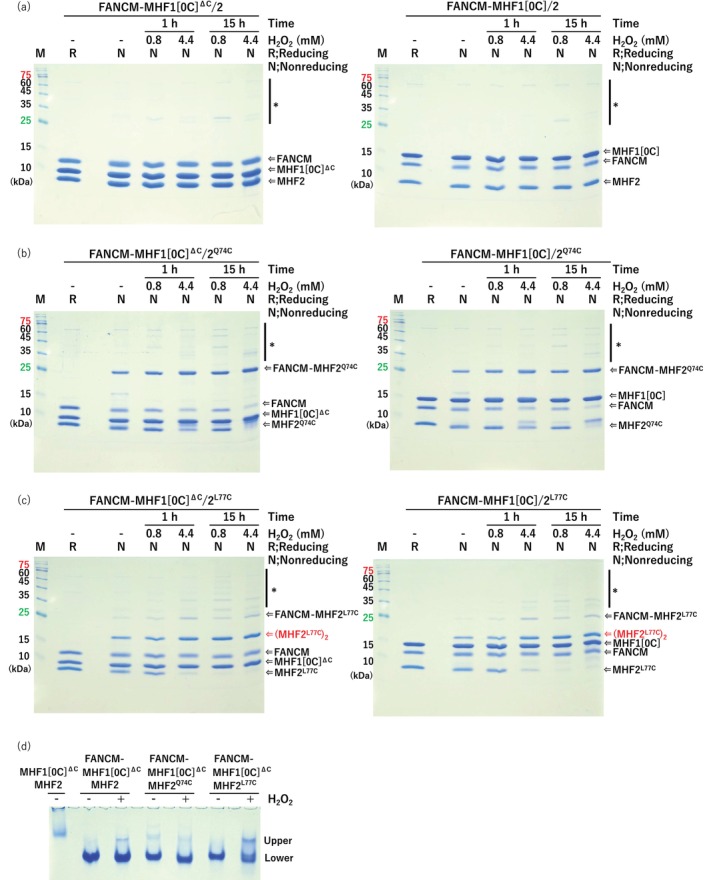
Disulfide crosslinking analysis of MHF2 variants. (a–c) Non‐reducing and reducing SDS‐PAGE of FANCM–MHF complexes after H_2_O_2_ treatment at indicated concentrations and time points, followed by *N*‐ethyl maleimide (NEM) alkylation. Left and right panels show results for complexes reconstituted with MHF1^ΔC^ and full‐length MHF1, respectively. (a) Non‐substituted control (FANCM–MHF[0C]). (b) Q74C variant; the FANCM–MHF2^Q74C^ crosslinked species (24.7 kDa) is the predominant product. (c) L77C variant; the MHF2^L77C^–MHF2^L77C^ homodimer (18.6 kDa, red arrowhead) is the predominant crosslinked species. In both Q74C and L77C variants, residual uncrosslinked MHF2 shows an upward band shift reflecting NEM adduct formation. R: Reducing; N: Non‐reducing; M: Molecular weight marker. Monomeric proteins: FANCM (15.4 kDa), MHF1[0C] full‐length (15.6 kDa), MHF1[0C]^ΔC^ (11.5 kDa), MHF2 (9.3 kDa). Bands above 30 kDa are indicated by an asterisk (*). The band at approximately 30 kDa likely arises from crosslinking involving endogenous FANCM Cys779. The band at approximately 70 kDa is attributed to a co‐purifying heat shock protein. Other bands in this region likely reflect intermolecular crosslinking among unreacted components. (d) Native PAGE analysis of MHF1[0C]^ΔC^/2, FANCM–MHF1[0C]^ΔC^/2, FANCM–MHF1[0C]^ΔC^/2^Q74C^, and FANCM–MHF1[0C]^ΔC^/2^L77C^ complexes in the absence (−) or presence (+) of H_2_O_2_. Upper and Lower bands correspond to FANCM‐associated histone fold (MHF) species and FANCM–MHF complex, respectively, as assigned based on previous studies (Ito & Nishino, 2021). Gels were stained with Coomassie Brilliant Blue R‐250.

To test whether the two designs stabilized the intended FANCM–MHF interface, we purified FANCM–MHF[0C] complexes containing either MHF2^Q74C^ or MHF2^L77C^ and subjected them to H_2_O_2_‐mediated oxidation, followed by *N*‐ethyl maleimide (NEM) alkylation to cap remaining free cysteines, and analyzed by non‐reducing SDS–‐PAGE (Figure 4a–c). Two MHF1 backgrounds were examined in parallel: full‐length MHF1 for DNA‐binding experiments, and the MHF1^ΔC^ construct lacking the C‐terminal disordered region for native PAGE and crystallization.

In the non‐substituted control (FANCM–MHF[0C]), a faint band at approximately 30 kDa was observed upon H_2_O_2_ treatment, likely arising from the endogenous FANCM Cys779, but no significant crosslinking was otherwise detected (Figure 4a). In contrast, both Q74C and L77C variants showed crosslinked species that accumulated in an H_2_O_2_ concentration‐ and time‐dependent manner, but with markedly different crosslinking patterns. In the Q74C variant, the intended FANCM–MHF2^Q74C^ crosslinked species was the predominant product, confirming selective stabilization of the FANCM–MHF2 interface (Figure 4b). In the L77C variant, the MHF2^L77C^‐MHF2^L77C^ homodimer was the predominant crosslinked species, whereas the intended FANCM–MHF2^L77C^ crosslinked band was minor, indicating that the competing MHF2‐MHF2 pathway dominated (Figure 4c).

In both Q74C and L77C variants, a residual population of uncrosslinked MHF2 was detected as an upward‐shifted band upon NEM treatment, confirming incomplete crosslinking in solution. The extent of the upward shift differed slightly between the two variants, likely reflecting differences in the number or accessibility of NEM‐reactive sites in each construct. In the full‐length MHF1 background, the NEM‐adducted MHF2 band was clearly resolved as a distinct band above the unmodified MHF2 band. In the MHF1^ΔC^ background, the NEM‐adducted MHF2 band was not directly visible as it co‐migrated with MHF1^ΔC^, but is presumed to be present based on the full‐length MHF1 results (Figure 4b,c).

To further assess complex integrity following oxidative crosslinking, native PAGE analysis was performed (Figure 4d). MHF[0C] alone produced a single band, whereas FANCM–MHF[0C] complexes showed two bands designated Upper and Lower. The Upper band migrated at a position comparable to that of MHF alone and is considered to correspond to a FANCM‐dissociated MHF species based on previous studies (Ito & Nishino, 2021). In FANCM–MHF[0C], H_2_O_2_ treatment resulted in an increase in the Upper band intensity, while the Lower band remained largely unchanged. In the Q74C variant, no significant change in either band was observed upon H_2_O_2_ treatment. In contrast, the L77C variant showed an increase in the Upper band and the appearance of the Lower band as a doublet upon H_2_O_2_ treatment, possibly reflecting the presence of multiple crosslinking states within the complex.

### 2.4 The Q74C design selectively stabilized the intact FANCM‐containing heteropentamer

**FIGURE 5:**
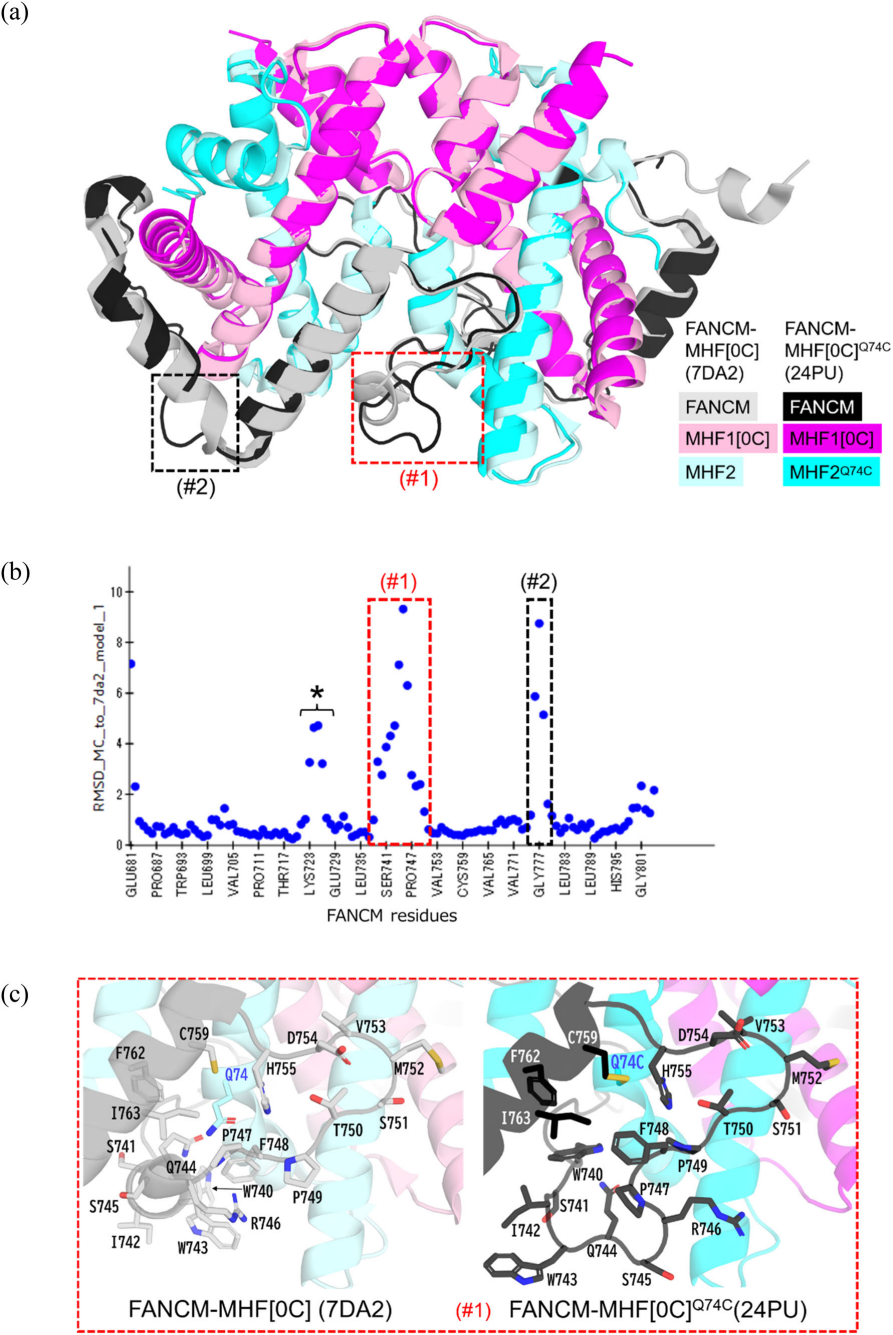
The Q74C design selectively stabilizes the intact FANCM‐containing heteropentamer. (a) Superposition of wild‐type FANCM–MHF[0C] structure (PDB ID: 7DA2) and the Q74C‐stabilized complex (PDB ID: 24PU). Pale colors indicate the wild‐type complex, whereas darker colors indicate the Q74C‐stabilized complex. FANCM is shown in gray/black, MHF1[0C] in pale magenta/magenta, MHF2 in pale cyan/cyan. The overall heteropentameric architecture is preserved in the Q74C‐stabilized complex. Regions exhibiting structural differences are highlighted by a red box (#1) and a black box (#2). (b) Per‐residue root‐mean‐square deviation plot for FANCM residues in the wild‐type and Q74C‐stabilized structures. The red box (#1) and black box (#2) indicate the same regions highlighted in (a). An asterisk indicates a region with weak electron density, corresponding to lower model reliability observed in both the wild‐type and Q74C crystal structures. (c) Detailed view of FANCM–MHF2 interface (Red box [#1] in a, b) in FANCM–MHF[0C] (left) and FANCM–MHF[0C]^Q74C^ (right). MHF2 Gln74/Cys74 and FANCM Cys759 are shown as sticks. Yellow bonds indicate disulfide.

To determine the structural consequence of the Q74C design, we solved the crystal structure of the oxidized FANCM–MHF[0C]^Q74C^ complex. The Q74C variant yielded crystals of the intact FANCM–MHF heteropentamer under MPD‐containing conditions. The Q74C structure was refined at 2.4 Å with Rwork and Rfree values of 0.2066 and 0.2748, respectively (Table 1), supporting reliable modeling of the engineered disulfide. Superposition with the wild‐type structure showed that the overall architecture of the complex was preserved (Figure 5a). Per‐residue root‐mean‐square deviation (r.m.s.d.) analysis indicated that structural differences were confined mainly to the region surrounding the engineered linkage (Figure 5b). Comparison with the wild‐type structure revealed modest structural deviations in two regions (Figure 5c). At the engineered crosslink site, cysteine substitution eliminated hydrogen bonds between MHF2 Gln74 and FANCM main‐chain atoms (Pro747 and Phe748). These changes lead to solvent exposure of Phe748 and disruption of the hydrophobic network with a nearby helix (Trp740 and Trp743) in the region proximal to the crosslink site, resulting in local helix unwinding (Figure 5a–c, red box #1). A secondary change was observed in a FANCM region exhibiting high *B*‐factors in the wild‐type structure (Figure 5a,b, black box #2), suggesting inherent conformational flexibility. Both changes occurred on the surface opposite to the DNA‐binding interface, and the core FANCM–MHF interface and histone‐fold architecture remained intact. Thus, Q74C selectively stabilized the intended FANCM–MHF2 interface without disrupting the overall heteropentameric architecture. The worldwide Protein Data Bank validation reports identified a small number of real‐space R‐value Z‐score outliers (24PU: D80, E723, C6, E775, and A13). These residues are located in surface‐exposed loop regions distant from both the engineered disulfide and the DNA‐binding interface, and do not affect the structural interpretations presented here. Complete crystallographic statistics are provided in Table 1.

**TABLE 1:** Data collection and refinement statistics.

Protein name	FANCM‐MHF[0C]^Q74C^	MHF[0C]^L77C^
(PDB ID)	(24PU)	(24PV)
Wavelength (Å)	0.98	0.98
Resolution range (Å)	46.48–2.4 (2.486–2.4)	42.49–4.0 (4.143–4.0)
Space group	P 21 21 21	C222_1_
Unit cell (Å)	59.527 90.452 92.955 90 90 90	80.794 86.583 221.329 90 90 90
Total reflections	131,798 (10294)	44,809 (4656)
Unique reflections	20,139 (1882)	6839 (663)
Multiplicity	6.5 (5.4)	6.6 (7.0)
Completeness (%)	99.45 (95.34)	99.33 (100.00)
Mean I/sigma (I)	8.87 (0.88)	10.35 (4.38)
Wilson *B*‐factor	48.79	104.70
R‐merge	0.155 (1.516)	0.1383 (0.483)
R‐meas	0.1685 (1.676)	0.1504 (0.5214)
R‐pim	0.0653 (0.6998)	0.05845 (0.1949)
CC1/2	0.996 (0.428)	0.995 (0.942)
CC*	0.999 (0.774)	0.999 (0.985)
Reflections used in refinement	20,129 (1883)	6815 (663)
Reflections used for Rfree	2003 (183)	686 (67)
Rwork	0.2066 (0.3612)	0.2582 (0.3295)
Rfree	0.2748 (0.3817)	0.3348 (0.4397)
CC (work)	0.964 (0.638)	0.924 (0.729)
CC (free)	0.917 (0.368)	0.879 (0.651)
Number of non‐hydrogen atoms	3878	5467
Macromolecules	3827	5467
Ligands	0	0
Solvent	51	0
Protein residues	473	686
RMS (bonds) (Å)	0.008	0.004
RMS (angles)	0.92	0.65
Ramachandran favored (%)	96.11	91.64
Ramachandran allowed (%)	3.46	7.46
Ramachandran outliers (%)	0.43	0.90
Rotamer outliers (%)	3.17	0.00
Clashscore	8.47	16.89
Average *B*‐factor	64.88	127.12
Macromolecules	65.01	127.12
Solvent	55.35	
Number of TLS groups	16	25

*Note*: Statistics for the highest‐resolution shell are shown in parentheses.

Abbreviation: MHF, FANCM‐associated histone fold; RMS, root‐mean‐square; TLS, translation libration screw.

### 2.5 The L77C design trapped an MHF tetramer through a competing MHF2‐MHF2 crosslinking pathway

**FIGURE 6:**
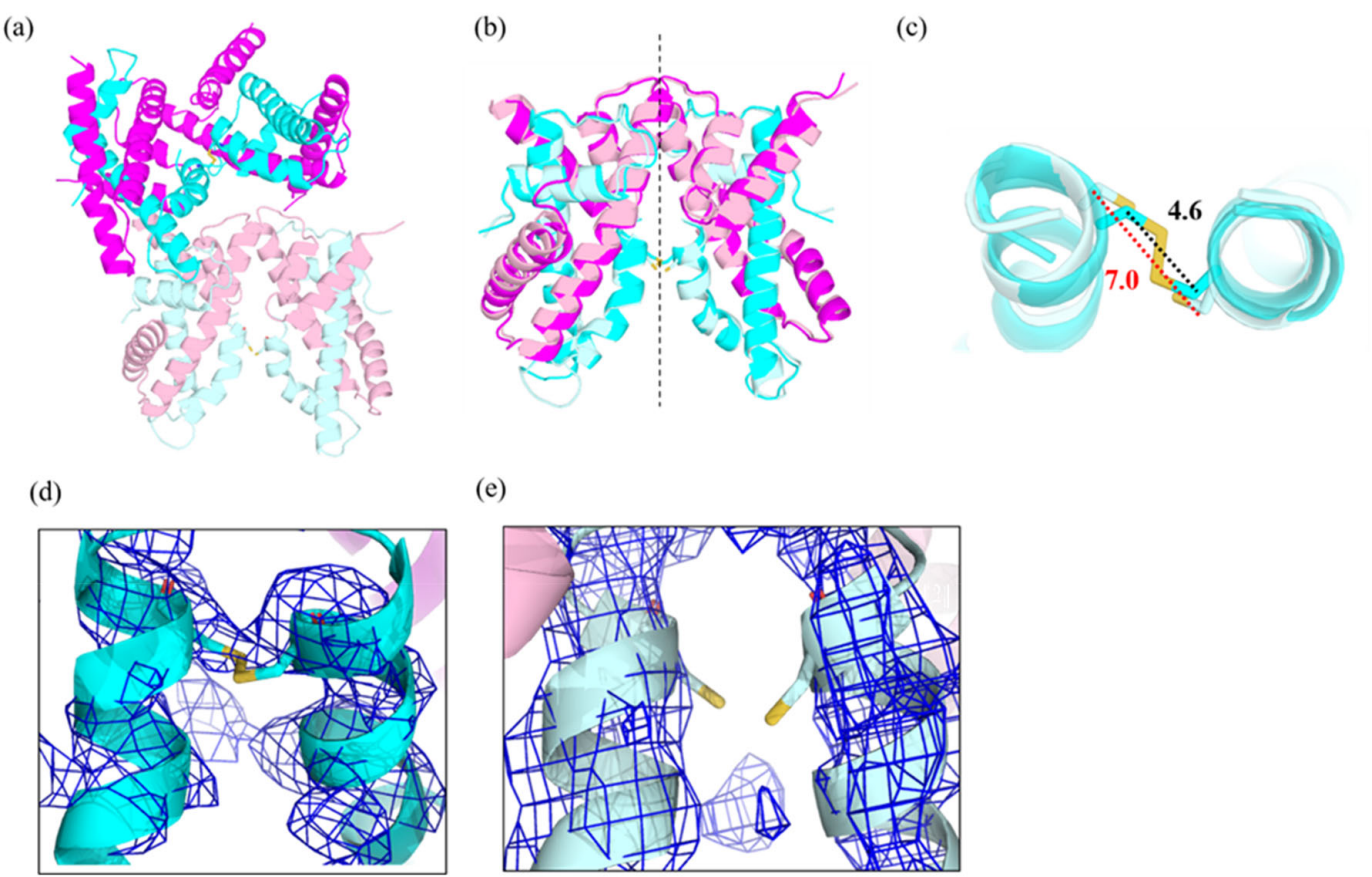
Crystal structure of MHF[0C]^L77C^ reveals competing MHF2‐MHF2 crosslinking in the asymmetric unit (ASU). (a) Crystal structure of the FANCM–MHF[0C]^L77C^‐derived sample. The ASU contains two crystallographically independent FANCM‐associated histone fold (MHF) heterotetramers, whereas FANCM is absent. The disulfide‐linked MHF[0C] tetramer is colored magenta (MHF1) and cyan (MHF2), while the non‐crosslinked tetramer is shown in pale magenta (MHF1) and pale cyan (MHF2), respectively. The side chains of residue L77C are shown as sticks, with sulfur atoms highlighted in yellow. The same color scheme as in (a) is used in panels (b–d). (b) Superposition of the two MHF heterotetramers shown in (a). The dashed line indicates the two‐fold symmetry axis of the MHF tetramer. (c) Close‐up view of residue 77 in the two tetramers, showing the Cβ–Cβ distance between the symmetry‐related Cys77 residues. One tetramer shows a short Cβ–Cβ distance (4.6 Å) compatible with disulfide formation, whereas the other shows a longer distance (7.0 Å) incompatible with crosslinking. (d) Simulated‐annealing omit map around the Cys77 pair in the disulfide‐linked tetramer, showing continuous density consistent with an MHF2^L77C^‐MHF2^L77C^ disulfide bond. Electron density shown in blue is contoured at 1*σ*. (e) Simulated‐annealing omit map around the Cys77 pair in the non‐crosslinked tetramer, showing no density consistent with disulfide formation. Electron density shown in blue is contoured at 1*σ*.

In contrast, the L77C variant yielded crystals containing only the MHF heterotetramer, indicating that FANCM dissociated during crystallization. The asymmetric unit (ASU) contained two crystallographically independent MHF tetramers (Figure 6a). The two tetramers superimposed closely overall (Figure 6b), with a main‐chain r.m.s.d. of 1.35 Å, but differed in the geometry around residue 77. One tetramer showed a short Cβ–Cβ distance between the symmetry‐related Cys77 residues, whereas the other showed a substantially longer distance (Figure 6c). Simulated‐annealing omit maps further supported distinct crosslinking states for the two tetramers: one showed continuous density consistent with an MHF2^L77C^‐MHF2^L77C^ disulfide bond (Figure 6d), whereas the other did not (Figure 6e). Comparison with the previously determined chicken MHF tetramer (PDB ID: 3B0B) yielded similarly low r.m.s.d. values for the non‐crosslinked and disulfide‐linked tetramers (1.58 and 1.75 Å, respectively), indicating that both retain an overall architecture comparable to that of the wild‐type assembly. At a local level, however, clear differences were observed around residue 77. Superposition revealed an approximately 1.9 Å displacement of the Cα atom at this position in the disulfide‐linked tetramer, and residue‐wise r.m.s.d. analysis showed deviations greater than 1.5 Å in the surrounding region (Figure S1a,b). Together, these data indicate that L77C favored a competing MHF2‐MHF2 linkage and trapped an MHF tetramer after FANCM dissociation, while also revealing local conformational heterogeneity within the MHF core. Data collection and refinement statistics are summarized in Table 1.

### 2.6 The Q74C‐stabilized complex retains FANCM–MHF‐specific DNA‐binding behavior

To assess whether the engineered disulfide affected DNA‐binding function, we performed electrophoretic mobility shift assays (EMSA) using the non‐substituted control construct (FANCM–MHF[0C]), and the Q74C and L77C variants, in the absence or presence of H_2_O_2_ or NEM treatment (Figure 3b).

On both the 49 bp duplex and HJ substrates, wild‐type FANCM–MHF and untreated FANCM–MHF[0C] produced characteristic FANCM‐MHF‐specific banding patterns (1# on 49 bp; 1# and 2# on HJ), distinct from those of MHF alone (1* and 2* on 49 bp; 1*–4* on HJ), confirming that the MHF1[0C] background does not alter DNA‐binding behavior (Figure 3a). Upon H_2_O_2_ treatment, FANCM‐MHF[0C] showed a shift toward MHF‐like banding patterns on both substrates, including the appearance of a 2* band on the 49 bp duplex and a shift toward 1*–4* on the HJ (Figure 3b, left). In contrast, the Q74C variant retained FANCM‐MHF‐specific banding patterns regardless of H_2_O_2_ or NEM treatment (Figure 3b, middle). The L77C variant retained FANCM‐MHF‐like binding in the untreated condition, but showed similar MHF‐like shifts upon either NEM treatment alone or H_2_O_2_ treatment (Figure 3b, right).

Taken together, these results demonstrate that the Q74C disulfide preserves FANCM‐MHF‐specific DNA‐binding behavior on both duplex and branched DNA substrates, consistent with the native PAGE results showing maintained complex integrity upon H_2_O_2_ treatment.

## 3 DISCUSSION

### 3.1 Disulfide engineering overcame a specific crystallization bottleneck in FANCM–MHF analysis

The main practical outcome of this study is that covalent stabilization can preserve the FANCM‐MHF complex under crystallization conditions that otherwise promote FANCM dissociation. Previous crystallization trials repeatedly yielded MHF‐containing crystals without FANCM, indicating that the FANCM–MHF interface is particularly vulnerable to amphiphilic precipitants such as MPD (Ito & Nishino, 2021, 2022). The Q74C design overcame this problem by selectively reinforcing the FANCM–MHF2 interface and enabling crystallization of the intact heteropentamer. Importantly, the Q74C‐stabilized complex retained FANCM–MHF‐specific complex integrity and DNA‐binding behavior under oxidative conditions, indicating that the engineered disulfide preserves a structurally and functionally relevant state rather than trapping a non‐native assembly. This advance is particularly relevant because the FANCM–MHF‐DNA ternary structure remains a major missing piece in the structural understanding of early FA‐pathway DNA recognition.

### 3.2 Geometric plausibility did not predict crosslinking specificity

The central conceptual finding of this study is that local geometric compatibility alone was insufficient to predict crosslinking specificity. Both Q74C and L77C were selected because they appeared compatible with disulfide formation with FANCM Cys759, and both underwent oxidation‐dependent crosslinking. However, only Q74C stabilized the intended FANCM–MHF2 interface, whereas L77C favored an alternative MHF2‐MHF2 linkage, indicating that the key issue was not whether crosslinking occurred, but which interface was preferentially trapped. This difference was directly reflected in complex integrity and functional behavior. Native PAGE analysis showed that the Q74C variant maintained complex integrity under oxidative conditions, whereas the L77C variant showed behavior consistent with FANCM dissociation. EMSA further demonstrated that the Q74C variant retained FANCM–MHF‐specific DNA‐binding behavior on both duplex and HJ substrates, whereas the L77C variant showed MHF‐like binding patterns under oxidative conditions and even upon NEM treatment alone, indicating particular sensitivity to steric perturbation at the L77C site.

This result exposes an important limitation of static structure‐based disulfide design. Current prediction methods are effective for identifying residue pairs with favorable local geometry (Craig & Dombkowski, 2013; Dani et al., 2003; Dombkowski, 2003), but they do not fully account for competing pathways created by oligomer symmetry. Our results therefore show that symmetry‐related competing pathways are a critical constraint on crosslink design in multimeric assemblies.

### 3.3 Constraints on crosslink design in symmetric oligomers

The present study demonstrates that geometric proximity of a target cysteine to both the intended partner and a symmetry‐related copy of the same residue creates a competing disulfide pathway that can dominate the intended reaction. Leu77 of MHF2 satisfies the geometric criteria for crosslinking with FANCM Cys759 (Sγ–Sγ distance of 3.9 Å), yet the proximity of the two‐fold‐symmetric Leu77 residues at the MHF2 dimer–dimer interface, where the Cγ–Cγ distance is 4.9 Å, provided an equally accessible competing acceptor. In contrast, Gln74 projects away from the symmetry‐related MHF2 subunit, placing the symmetry‐related Cα more than 9 Å apart and effectively eliminating this competing pathway. The controlled comparison between Q74C and L77C where two designs sharing the same endogenous anchor (Cys759) and differing primarily in their spatial relationship to the MHF2 dimer–dimer axis, isolates the contribution of symmetry‐related competing pathways as a design variable.

The principle revealed here has clear parallels in the literature. The H2A‐N38C nucleosome (Frouws et al., 2018) provides a striking example in which the same symmetry‐driven reactivity was instead the design goal: engineered cysteines at the dyad‐related H2A position 38 (Cα–Cα distance of 5.5 Å), whose side chains already face each other across the two‐fold axis, successfully formed an inter‐subunit disulfide that stabilized the histone octamer against H2A/H2B dimer dissociation. Similarly, dimer‐interface disulfides have been intentionally engineered into the homodimeric thymidylate synthase (Agarwalla et al., 1996; Gokhale et al., 1994; Velanker et al., 1999) and incorporated into synthetic‐symmetrization strategies for crystallization (Banatao et al., 2006), in the latter case with explicit removal of native cysteines to avoid unintended inter‐subunit reactivity. Off‐target disulfide formation with symmetry‐related residues has also been recognized as an artifact in disulfide trapping of receptor–ligand complexes (Kufareva et al., 2016). What distinguishes the present L77C case is a direct structural demonstration that, within a single oligomeric architecture and using the same FANCM anchor (Cys759), two closely related MHF2 designs lead to fundamentally different outcomes, establishing the spatial relationship to the symmetry‐related subunit as a criterion that must be evaluated independently of local geometry with the intended partner.

### 3.4 The L77C structure reveals plasticity within the MHF tetramer

Although L77C did not preserve the FANCM‐containing heteropentamer, it still provided important structural information. The presence of one crosslinked and one non‐crosslinked MHF tetramer in the same ASU indicates that the MHF dimer–dimer interface can adopt distinct local states, only one of which is competent for disulfide formation at position 77. This observation is consistent with the view that the MHF tetramer is structurally plastic rather than rigid. Analysis of crystal contacts using PDBePISA indicated that the region surrounding residue 77 participates in intratetrameric subunit–subunit interactions within the MHF assembly but does not make direct crystal‐packing contacts. The observed heterogeneity therefore most likely reflects intrinsic conformational differences at the dimer–dimer interface rather than a crystal‐packing artifact. This interpretation is also consistent with the biochemical observation that L77C does not channel the reaction cleanly into a single crosslinked state, as evidenced by the appearance of the Lower band as a doublet in native PAGE upon H_2_O_2_ treatment.

The functional sensitivity of the L77C site was further highlighted by the observation that NEM alkylation alone, without H_2_O_2_‐mediated oxidation, was sufficient to shift the DNA‐binding pattern of the L77C variant toward that of MHF alone on both duplex and HJ substrates. This effect was not observed with Q74C under equivalent conditions, indicating that it is specific to the L77C site rather than a general consequence of cysteine alkylation. One interpretation is that NEM adducts at both FANCM Cys759 and MHF2 Cys77, which lie in close proximity at the FANCM–MHF2 interface, introduce sufficient steric bulk to destabilize the interface and promote FANCM dissociation. This positional sensitivity suggests that the region around Leu77 plays a role in the dynamic regulation of FANCM–MHF association, and that even modest steric perturbation at this site can tip the equilibrium toward dissociation.

More generally, the L77C result shows that an apparently unsuccessful crosslink design can still reveal alternative assembly states that are otherwise difficult to observe. The broader relevance of this plasticity is supported by the known behavior of CENP‐S/X‐related histone‐fold assemblies, in which the CENP‐SX dimer associates with the centromere protein T‐W (CENP‐TW) dimer to form the CENP‐TWSX heterotetramer (Nishino et al., 2012). Formation of CENP‐TWSX requires dissociation of the (CENP‐S‐CENP‐X)_2_ tetramer into heterodimers, implying that the ability to adopt a dissociation‐competent state is functionally required.

### 3.5 Applicability beyond the chicken FANCM–MHF complex

The present study uses the chicken FANCM–MHF system, and the specific Q74C strategy is not expected to transfer directly to the human complex because the corresponding region differs in sequence and, in human FANCM, the equivalent cysteine contributes to zinc coordination (Fox et al., 2014; Ito & Nishino, 2021). Nevertheless, the underlying design principle remains general: an endogenous or minimally perturbing cysteine anchor can be paired with a complementary cysteine on the partner subunit, provided that competing symmetry‐related pathways are explicitly considered during design. More broadly, this constraint is likely to apply to many multimeric systems with internal symmetry, including histone‐fold assemblies and other oligomers in which one engineered cysteine can access more than one geometrically plausible reaction partner.

## 4 CONCLUSION

Disulfide engineering enabled stabilization of the FANCM–MHF complex under crystallization conditions that otherwise cause FANCM dissociation. Using the endogenous FANCM Cys759 as a covalent anchor, two candidate designs were evaluated: Q74C selectively preserved the intended FANCM‐containing heteropentamer, whereas L77C was diverted into a competing MHF2‐MHF2 pathway and trapped an MHF tetramer. Biochemical and functional analyses further showed that the Q74C‐stabilized complex maintains FANCM–MHF‐specific complex integrity and DNA‐binding behavior under oxidative conditions, whereas the L77C variant is sensitive to steric perturbation at the FANCM–MHF2 interface. Structural analysis, supported by crystallographic validation of the L77C crosslinking states and complete refinement statistics for both structures, shows that crosslinking specificity in symmetric oligomers cannot be predicted from local geometry alone, but must also account for symmetry‐related competing pathways. This study therefore provides both a practical stabilization strategy for FANCM–MHF and a general design constraint for engineered disulfides in multimeric protein assemblies.

## 5 MATERIALS AND METHODS

### 5.1 FANCM construct optimization

The FANCM construct was refined to residues 680–804 based on the crystal structure (PDB entry 7DA2), removing the disordered N‐terminal region (residues 660–679) that was not resolved in electron density maps. After tobacco etch virus (TEV) protease cleavage of the MBP‐His_6_ tag, vector‐derived residues GRA (N‐terminus; G from TEV recognition site, RA from pRSFDuet‐1 linker) and P (C‐terminus; from pRSFDuet‐1 linker) remain, yielding the final construct GRA‐FANCM (680–804)‐P. Removal of residues 660–679 has the additional benefit of eliminating two endogenous cysteines (Cys670 and Cys679), leaving two endogenous cysteines, Cys759 and Cys779, within the FANCM construct. Throughout this manuscript, “FANCM” refers to residues 680–804 unless otherwise noted.

### 5.2 Disulfide bond design

Candidate residues for disulfide engineering were identified by structure‐based analysis of the FANCM–(MHF1^ΔC^‐MHF2)_2_ crystal structure (PDB entry 7DA2; Ito & Nishino, 2021) using Discovery Studio 2021 (BIOVIA, Dassault Systèmes). The optimized FANCM construct (residues 680–804) contains two endogenous cysteines: Cys759, located at the FANCM–MHF2 interface, and Cys779, which is positioned on the opposite face of FANCM with the nearest MHF residue being MHF1 Lys44 (Sγ–Cγ distance: 5.9 Å). Because Cys759 was the only endogenous cysteine at a protein–protein interface with favorable proximity to a potential partner residue, it was selected as the anchor point for intermolecular disulfide crosslinking. Three MHF2 residues within 4 Å of FANCM Cys759 were identified by manual inspection: Gln74, Leu77, and Asp78. Each residue was computationally mutated to cysteine using the Build Mutants tool in Discovery Studio (Macromolecules > Design Protein > Build Mutants), which employs MODELER (Sali & Blundell, 1993) to optimize the conformation of mutated residues and neighboring atoms. For each mutation, only the mutated residue was optimized (Cut Radius = 0 Å) as the substitutions involved residues of similar size. The geometric feasibility of disulfide bond formation with Cys759 was evaluated based on Cβ–Cβ distance, dihedral angle compatibility, and absence of steric clashes. MHF2^Q74C^ exhibited favorable geometry for disulfide formation, while MHF2^D78C^ showed excessive strain in both distance and angle parameters and was excluded from experimental testing. Based on these geometric evaluations, MHF2^Q74C^/FANCM^C759^ and MHF2^L77C^/FANCM^C759^ were selected for experimental validation.

### 5.3 Protein expression and purification

#### 5.3.1 Expression constructs and mutagenesis

All proteins were derived from chicken (*Gallus gallus*) sequences. For co‐expression of FANCM and the MHF complex, the FANCM gene (including the tac promoter and an N‐terminal MBP‐His_6_‐TEV tag) was subcloned from the original pMal‐based vector into pRSFDuet‐1 (Novagen) downstream of MHF2. The resulting vector encodes MHF1 (His_6_‐TEV‐MHF1, residues 2–139 or MHF1^ΔC^, residues 2–106) and MHF2 (Strep‐TEV‐MHF2, residues 2–80) under T7 promoters, and FANCM (MBP‐His_6_‐TEV‐FANCM, residues 680–804) under the tac promoter. Except for the initial crystallization trials with DNA substrates described in Section 2.1, all experiments were performed using an MHF1 variant in which the three endogenous cysteine residues (Cys26, Cys28, and Cys55) were substituted with alanine (MHF1[0C]), as described previously (Ito & Nishino, 2021, 2022). Kanamycin selection provided stable selective pressure and improved plasmid stability compared to ampicillin, resulting in consistent protein expression levels. This pRSFDuet‐1 construct was used as the template for site‐directed mutagenesis, and MHF2 mutations (Q74C and L77C) were introduced using the QuikChange protocol with PrimeSTAR Max DNA Polymerase (Takara Bio).

#### 5.3.2 Protein expression

*Escherichia coli* BL21 Star (DE3) pLysS with RARE2 transfer RNA supplement (Agilent Technologies) cells transformed with the expression vector were cultured in Luria–Bertani (broth) medium containing kanamycin (50 μg/mL) at 37°C to optical density_600_ = 0.6, induced with 0.5 mM isopropyl β‐D‐1‐thiogalactopyranoside, and grown overnight at 20°C.

#### 5.3.3 Protein purification

Harvested cells were resuspended in lysis buffer (10 mM Tris–HCl pH 8.0, 500 mM NaCl) and lysed by sonication. The lysate was treated with 0.1% (*w*/*v*) polyethylenimine (PEI) to precipitate nucleic acids, clarified by centrifugation, and filtered through a 0.45 μm filter. The clarified lysate was applied to a HisTrap FF column (Cytiva) pre‐equilibrated in purification buffer (10 mM Tris–HCl pH 8.0, 500 mM NaCl, 25 mM imidazole). The column was washed extensively with purification buffer and eluted with elution buffer (10 mM Tris–HCl pH 8.0, 500 mM NaCl, and 500 mM imidazole). The eluate was desalted using a HiPrep 26/10 Desalting column (Bio‐Rad) equilibrated in desalting buffer (10 mM Tris–HCl pH 8.0, 500 mM NaCl). The N‐terminal tags were cleaved by his‐TEV protease overnight at room temperature. The cleaved complex was applied to tandem MBPTrap HP and HisTrap FF columns (Cytiva) to remove uncleaved protein, free tags, and his‐TEV protease. The flow‐through fraction was concentrated using Amicon Ultra centrifugal filters (Merck Millipore) and further purified by gel filtration on a Superdex 200 16/600 PG column (Cytiva) equilibrated in storage buffer (10 mM Tris–HCl pH 8.0, 500 mM NaCl). Peak fractions were concentrated (determined by *A*_280_ using a NanoDrop spectrophotometer) and stored at −80°C.

### 5.4 Disulfide crosslinking analysis

Purified FANCM–MHF complexes (non‐substituted control, Q74C, or L77C) at 1.2 mg/mL (approximately 18–20 μM) in storage buffer were oxidized with H_2_O_2_ at a final concentration of 0.8 mM (approximately 40‐fold molar excess) or 4.4 mM (approximately 220‐fold molar excess) at 4°C for 1 or 15 h. Following oxidation, free cysteines were capped by addition of NEM to a final concentration of 5 mM and incubated for 15 min at room temperature. For non‐reducing SDS‐PAGE analysis, samples were mixed with SDS‐PAGE sample buffer without reducing agents and heated at 95°C for 5 min. For reducing SDS‐PAGE, samples were supplemented with 5% 2‐mercaptoethanol before heating. Samples were analyzed by 14% SDS‐PAGE and visualized by Coomassie Brilliant Blue R‐250 staining.

### 5.5 Native PAGE analysis

Native PAGE was performed to assess complex integrity and electrophoretic mobility. Protein samples were mixed with native PAGE sample buffer (final: 10 mM Tris–HCl pH 8.0, 500 mM NaCl, 10% glycerol, and 0.01% bromophenol blue). To test stability in the presence of precipitant, samples (1.5 mg/mL) were supplemented with MPD to final concentrations of 0–30% (*v*/*v*) and incubated at 4°C overnight before electrophoresis. For assessment of complex integrity following oxidative crosslinking, aliquots of the samples treated with 4.4 mM H_2_O_2_ as described in Section 5.4, prior to NEM treatment, were analyzed by native PAGE. Samples were electrophoresed on 6% polyacrylamide gels in 0.5× TBE buffer (pH 8.0) at 4°C and 120 V for 120 min. Gels were stained with Coomassie Brilliant Blue R‐250.

### 5.6 Crystallization

The concentrated protein samples at 10–11 mg/mL were treated with 0.1% H_2_O_2_ (approximately 150‐fold molar excess) at 4°C overnight. Oxidized FANCM–MHF complexes were crystallized by the sitting‐drop vapor diffusion method at 20°C. Crystallization conditions were optimized based on those used for the FANCM–MHF complex (Ito & Nishino, 2021). For the FANCM–MHF^Q74C^ complex, protein at 11 mg/mL in 10 mM Tris–HCl pH 8.0, 500 mM NaCl was mixed with reservoir solution containing 0.1M Tris‐Bicine pH 8.7, 50 mM carboxylic acid mix, 15% MPD, 20% polyethylene glycol (PEG) 3350, and 100 mM NaCl. For the MHF2^L77C^ variant, protein at 10 mg/mL in 10 mM Tris–HCl pH 8.0, 250 mM NaCl was mixed with reservoir solution containing 0.1 M Tris‐Bicine pH 8.8, 0.1M carboxylic acid mix, 14% MPD, 20.5% PEG 3350, and 250 mM NaCl. Drops were prepared by mixing 0.8 μL protein solution with 0.8 μL reservoir solution and equilibrated against 50 μL reservoir solution. Crystals appeared within approximately 1 week and grew to 0.1–0.2 mm. Crystals were cryoprotected by supplementing reservoir solution with 3% PEG 3350 and 2% glycerol, equilibrating for 90 min, and flash‐cooling in liquid nitrogen.

### 5.7 Data collection and structure determination

X‐ray diffraction data were collected at beamline BL‐17A of the Photon Factory (Tsukuba, Japan) at 100 K using an EIGER detector (4150 × 4371 pixels, 0.075 mm pixel size). Data were collected with a wavelength of 0.98 Å, a detector distance of 390 mm, and an oscillation range of 0.5° per frame over a total rotation of 180° (360 frames). Data were processed with X‐ray Detector Software (Kabsch, 2010), and space group determination and scaling were performed with POINTLESS and AIMLESS from the CCP4 suite (Evans & Murshudov, 2013; Winn et al., 2011).

The structures were solved by molecular replacement using Phaser (McCoy, 2007) within the PHENIX suite (Liebschner et al., 2019). For the FmMHF^Q74C^ structure, the search model was the wild‐type FANCM–MHF heteropentamer structure (PDB entry 7DA2; Ito & Nishino, 2021). For the MHF^L77C^ structure, the search model was the MHF heterotetramer (PDB entry 3B0B; Nishino et al., 2012). Model building and refinement were performed iteratively using Coot (Emsley et al., 2010) and PHENIX. The engineered disulfide bonds were modeled based on clear continuous electron density in 2Fo‐Fc and Fo‐Fc maps. The final models were validated using MolProbity (Chen et al., 2010). For the 4.0 Å MHF^L77C^ structure, refinement employed tighter geometric restraints, group *B*‐factor refinement, and 25 translation libration screw groups; no water molecules were modeled. Data collection and refinement statistics are summarized in Table 1.

### 5.8 Interface and crystal‐packing analysis

Crystal‐packing analysis was performed using PDBePISA to examine whether the region surrounding MHF2 Cys77 participates in crystal contacts or instead reflects intratetrameric subunit–subunit interactions within the MHF assembly (Krissinel & Henrick, 2007). This analysis was used to support interpretation of the distinct crosslinking states observed for the two tetramers in the ASU.

### 5.9 DNA substrates preparation

DNA oligonucleotides were purchased from Eurofins Genomics and used without further purification. For double‐stranded DNA substrates and HJ substrates, a 49 bp duplex derived from the Widom 601 nucleosome positioning sequence was prepared by annealing two complementary 49‐nt oligonucleotides, and HJ substrates were prepared by annealing four 50‐nt oligonucleotides (Table S1). Complementary oligonucleotides were annealed in Tris‐EDTA buffer (10 mM Tris–HCl pH 8.0, 1 mM ethylenediaminetetraacetic acid) by heating to 95°C for 5 min followed by slow cooling to 4°C over 60 min.

### 5.10 Electrophoretic mobility shift assays

Binding reactions were prepared by mixing 4 μL of protein solution with 4 μL of DNA substrate (final DNA concentration 0.4 μM) in binding buffer (10 mM Tris–HCl pH 8.0, 150 mM NaCl) at protein‐to‐DNA molar ratios of 0.66×, 1.33×, 1.99×, 2.66×, 3.33×, and 3.99×. Reactions were incubated at room temperature for 10 min, then 2 μL of loading dye (50% sucrose containing UltraPower Nucleic Acid Stain [Gellex International, Machida, Japan] and Orange G) was added and 5 μL was applied to the gel. Samples were analyzed by native polyacrylamide gel electrophoresis (6% gels) in 0.5× Tris‐borate‐EDTA (TBE) buffer at 4°C. The 49 bp duplex substrate was electrophoresed at 120 V for 90 min, and the HJ substrate was electrophoresed at 120 V for 180 min. DNA was visualized by UV fluorescence using a gel imaging system.

## AUTHOR CONTRIBUTIONS

**Sho Ito**: Conceptualization, Methodology, Investigation, Formal analysis, Writing—original draft. **Tatsuya Nishino**: Conceptualization, Supervision, Funding acquisition, Writing—review & editing.

## CONFLICT OF INTEREST STATEMENT

The authors declare no conflict of interest.

## Supporting information


**Data S1.** Supporting Information.

## Data Availability

The atomic coordinates and structure factors for the FANCM–MHF^Q74C^ and FANCM–MHF^L77C^ crystal structures have been deposited in the Protein Data Bank under accession codes 24PU and 24PV, respectively.
