# Supplementary material for: Disulfide engineering of the FANCM–MHF complex reveals constraints on crosslink design in symmetric oligomers
Source: Protein Sci. 2026 Aug 17;35(9):e70761. doi: 10.1002/pro.70761 (PMC13479805; doi:10.1002/pro.70761)
Supplement: Supplementary file 1 [file PRO-35-e70761-s001.docx]

**Supplementary Figure and Table Legend**

(a)

(b)

Supplementary Figure S1: (a) Superposition of three MHF structures: MHF^WT^ and the two molecules in the asymmetric unit (ASU) of MHF^L77C^. MHF^WT^ is shown in gray, the non‑crosslinked molecule (Non‑XL) of MHF^L77C^ in blue, and the crosslinked molecule (XL) in green. The right panel presents an enlarged top view of the crosslinking region, showing residues L76, L77, and D78. (b) Residue‑wise main‑chain (MC) RMSD analysis of the 24PV‑2 crosslinked structure (XL) to MHF^WT^ (gray) and the Non‑XL molecule (blue). The plots show chain‑specific RMSD values, while the table summarizes the number and percentage of residues with RMSD > 1.5 Å. Asterisks (*) indicate residues proximal to the crosslink.

**Supplementary Table S1:** Oligonucleotide sequences used for DNA substrate preparation.

| **Substrate** | **Name** | **Sequence (5'→3')** | **Length (nt)** |
| --- | --- | --- | --- |
| 49 bp dsDNA | Widom-601-49-1 | GCACCGCTTAAACGCACGTACGCGCTGTCCCCCGCGTTTTAACCGCCAA | 49 |
|  | Widom-601-49-2 | TTGGCGGTTAAAACGCGGGGGACAGCGCGTACGTGCGTTTAAGCGGTGC | 49 |
| HJ2 | HJ2-1 | GGTAGGACGGCCTCGCAATCGGCATTCACCGAGCACGCGAGATGTCAACG | 50 |
|  | HJ2-2 | CGTTGACATCTCGCGTGCTCGGTGAATCGGCAGATGCGGAGTGAAGTTCC | 50 |
|  | HJ2-3 | GGAACTTCACTCCGCATCTGCCGATTGTGGCTGTGGCGTGTTTCTGGTGG | 50 |
|  | HJ2-4 | CCACCAGAAACACGCCACAGCCACAATGCCGATTGCGAGGCCGTCCTACC | 50 |
